# Investigating cat predation as the cause of bat wing tears using forensic DNA analysis

**DOI:** 10.1002/ece3.6544

**Published:** 2020-07-06

**Authors:** Rana O. S. Khayat, Robyn A. Grant, Hazel Ryan, Louise M. Melling, Gary Dougill, David R. Killick, Kirsty J. Shaw

**Affiliations:** ^1^ Faculty of Science and Engineering Manchester Metropolitan University Manchester UK; ^2^ Department of Biology Umm Al‐Qura University Makkah Saudi Arabia; ^3^ The Wildwood Trust Herne Common UK; ^4^ Institute of Infection, Veterinary and Ecological Sciences University of Liverpool, Leahurst Liverpool UK

**Keywords:** bat, cat, predation, STR analysis

## Abstract

Cat predation upon bat species has been reported to have significant effects on bat populations in both rural and urban areas. The majority of research in this area has focussed on observational data from bat rehabilitators documenting injuries, and cat owners, when domestic cats present prey. However, this has the potential to underestimate the number of bats killed or injured by cats. Here, we use forensic DNA analysis techniques to analyze swabs taken from injured bats in the United Kingdom, mainly including *Pipistrellus pipistrellus* (40 out of 72 specimens). Using quantitative PCR, cat DNA was found in two‐thirds of samples submitted by bat rehabilitators. Of these samples, short tandem repeat analysis produced partial DNA profiles for approximately one‐third of samples, which could be used to link predation events to individual cats. The use of genetic analysis can complement observational data and potentially provide additional information to give a more accurate estimation of cat predation.

## Graphical abstract

Forensic DNA analysis techniques were used to analyze swabs taken from injured bats in the United Kingdom. Cat DNA was found to be present in two‐thirds of samples, and of these samples, short tandem repeat analysis produced partial DNA profiles for approximately one‐third of samples, which could be used to link predation events to individual cats. The use of genetic analysis can complement observational data and potentially provide additional information to give a more accurate estimation of cat predation.

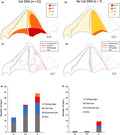

## 1 INTRODUCTION

Bats play an important role in many ecosystems, through pest control, pollination, and/or seed dispersal, and can be found across the world in both rural and urban areas. Urban expansion has had a significant effect on bat populations, with a highly species‐specific response (Jung & Threlfall, 2018). Urbanization can offer advantages in terms of increased roosting and foraging opportunities, but also disadvantages as a result of the loss or fragmentation of key natural habitats, exposure to urban predators, and physical, chemical, or light pollution (Russo & Ancillotto, 2015). This rapid environmental change can also produce ecological traps, that is, scenarios where the bats will settle in poor‐quality habitats or maintain roost fidelity despite alterations to the environment. For example, high mortality rates of common noctule bats (*Nyctalus noctula*) were observed in one concrete building in Kharkiv City, Ukraine, with cat predation reported as the most common cause (157 out of 231 deaths), compared to starvation, dehydration, chemical contamination or being killed by humans (Vlaschenko, Kovalov, Hukov, Kravchenko, & Rodenko, 2019).

The presence of predators in urban areas, such as domestic cats (*Felis catus*), can strongly influence roost selection by bats and can even cause them to abandon roosts altogether (Welch & Leppanen, 2017). It has been hypothesized that the cats are attracted to the bat roosts by sensory cues, including sound, smell, and vision (Ancillotto, Serangeli, & Russo, 2013). The hunting strategy of feral cats has been observed using infrared cameras in the Culebrones Cave in Puerto Rico, which is home to ~300,000 individual bats. It showed that cats hunted either by sitting on their hind legs and catching bats in the air with a swift movement of their paws, or by jumping and catching bats in mid‐air (Rodríguez‐Durán, Pérez, Montalbán, & Sandoval, 2010), resulting in either death or injury to the bat.

In the United Kingdom (UK), many injured or orphaned bats, mostly common pipistrelles (*Pipistrellus pipistrellus*) and soprano pipistrelles (*Pipistrellus pygmaeus*), are cared for by specialist bat rehabilitators or general wildlife rehabilitation centers. For example, the Royal Society for the Prevention of Cruelty to Animals (RSPCA) at Stapeley Grange Wildlife Centre in North West England admitted 748 pipistrelles (*Pipistrellus* spp.) over a 10 year period from 1997 to 2006 (Kelly, Goodwin, Grogan, & Mathews, 2008). A survey of bat rehabilitators around the UK estimated that 2,000 bats, specifically with wing tear injuries, are taken to rescue centers for rehabilitation annually in the UK, which especially affects the most abundant common pipistrelle.

Wing tears are commonly encountered injuries in bats, but can have significant consequences, particularly with regard to flight (Voigt, 2013). Rehabilitation of bats from wing tears is possible since the wings have an extensive blood supply that enables wound cleaning, infection prevention, and tissue reformation (Faure, Re, & Clare, 2009). We have recently shown that most tears in bat wings occur in the plagiopatagium, the most proximal wing section to the body (Khayat et al., 2019). We suggested that predator attacks were the likely cause of many of the tears, with directed attacks to the body resulting in many of the rostro‐caudal tears observed in the plagiopatagium (Khayat et al., 2019).

The majority of research into cat attacks on bat species has been based on observational data. As cats often present their kills to their owners, it allows these observations to be recorded (Welch & Leppanen, 2017). Single kills of species of conservation concern have been reported globally, for example, a female adult domestic cat bringing home an endangered Madeira pipistrelle (*Pipistrellus maderensis*) in Madeira (Rocha, 2015), an Eastern blossom bat (*Syconycteris australis*) being brought to a farmer's house in Australia (Phillips, Coburn, & James, 2001) and a farmer's cat preying on a Ryukyu flying fox (*Pteropus dasymallus*), listed as vulnerable, in Japan (Vincenot, Koyama, & Russo, 2015). Larger studies have surveyed cat owners to monitor prey deposition at owner's residences. In Canberra, Australia, a year‐long study which recorded a total of 1,961 prey from 214 cats found only five bats (0.25%) (Barratt, 1997), which agrees with a study in the UK which showed that 30 out of 9,852 (0.3%) of mammals brought in by cats were bats (Woods, McDonald, & Harris, 2003), with lower bat predation rates of just 0.06% recorded in mainland southwest Finland (Kauhala, Talvitie, & Vuorisalo, 2015). In some reports, the reason for assigning bat deaths due to cat predation is not clearly defined. Examples include a study in New Zealand which attributed 40% of deaths of long‐tailed bats (*Chalinolobus tuberculatus*) and short‐tailed bats (*Mystacina* spp.) to cat predation (Daniel & Williams, 1984), and a report from Italy which analyzed 1,012 records of bats admitted to four rescue centers from 2009 to 2011 and reported cat predation as the contributing factor in 28.7% of cases (Ancillotto et al., 2013).

Forensic analysis to investigate cat predation on bat species has received limited attention in the literature and has focussed primarily on postmortem examination. In a study of 486 deceased bats, comprising 19 European species including *Pipistrellus*, 39% had sustained mild to severe trauma. Although the reasons for these causes of death were not explicitly given, it was estimated that cat predation was responsible for almost half of these cases, most commonly wing membrane lacerations and soft tissue damage, followed by wound infection with the bacteria *Pasteurella multocida*, which is the most common cause of infection of the chest cavity (pyothorax) in cats and can be transferred upon interaction (Mühldorfer, Speck, & Wibbelt, 2011; Walker, Jang, & Hirsh, 2000). A study on *Mystacina* spp. in New Zealand observed extensive predation on a single roost, with 156 individual bat wings and 22 intact bodies found in just 1 week. Seven of the bats were sent for postmortem analysis: two had clear puncture wounds and five had tearing, bruising, and internal bleeding. Examination of the puncture wounds allowed calculation of the intercanine dimensions of the predator, indicative of a cat or large ferret. DNA analysis was carried out on hair strands found in the roost and these matched a male cat caught at the bottom of the tree, although no details on the analysis were provided. No further bat predation was observed following the capture of the male cat (Scrimgeour, Beath, & Swanney, 2012). Cases such as this highlight the concept of prey specialization in cats, with both domestic (Dickman & Newsome, 2015) and feral (Moseby, Peacock, & Read, 2015) cats having been found to show individual preferences for different prey species.

Forensic DNA analysis predominantly focusses on the use of short tandem repeat (STR) markers for human identification purposes, within a legal framework for civil or criminal cases. However, application of similar principles to analysis of non‐human DNA sources has also been demonstrated, particularly for domestic animals such as dogs and cats, where the animal can be viewed as a potential victim, perpetrator, or witness to a crime (Butler, 2010). A multiplex STR system, known as the “Meowplex,” has been developed which enables genetic individualization of domestic cats through analysis of eleven STR loci and a sex marker on the Y chromosome, SRY (Menotti‐Raymond, David, Stephens, Lyons, & O'Brien, 1997; Menotti‐Raymond, David, Wachter, Butler, & O'Brien, 2005). This principle has been successfully used in forensic cases, for example, by matching the DNA profile of a cat hair found on an item of evidence linked to a homicide to that of the suspect's pet cat, Snowball (Menotti‐Raymond, David, & O'Brien, 1997).

Here, we demonstrate how established forensic DNA analysis can be applied to detect the presence of cat DNA on bat wing traumas and produce individual cat DNA profiles for identification purposes. The genetic data were then compared to information collected from bat rehabilitators on (a) bat characteristics, that is, species, age, gender, and location; (b) wing tear characteristics and placement; and (c) suspected cause of wing tear injuries.

## 2 MATERIALS AND METHODS

### 2.1 Biological sample collection

Ethical approval was obtained through the Research Ethics and Governance Committee at Manchester Metropolitan University (Reference Number 1255). Cat blood samples (07906, 00932, and 01606), used as positive controls for all genetic experiments, were collected in sample vials containing an anticoagulant (ethylenediaminetetraacetic acid (EDTA)), and were surplus after use for clinical tests at the Institute of Infection, Veterinary and Ecological Sciences, University of Liverpool (University of Liverpool ethical approval VREC656).

Bat wing swab samples were obtained from bat rehabilitators in the UK. Bat rehabilitators were all trained individuals and registered with the Bat Conservation Trust (BCT). Rehabilitators were recruited by advertising the project at the Mammal Society Meetings, the National Bat Conference, the National Bat Care Conference and in “Bat Care News,” as well as on Facebook groups across the UK (UK Bat Workers, Cambridgeshire Bat Group, Kent Bat Group, and South Lancashire Bat Group). Samples were collected between March 2016 and September 2018, either from live, rehabilitating animals or those that were badly injured and required euthanasia (Miller, 2016). Bat rehabilitators were asked to wear a clean pair of nitrile gloves and swab the site of any wing injuries on bats as soon as they arrived in their care, following detailed instructions provided that were in agreement with the Bat Conservation Trust Bat Care Guidelines (Miller, 2016). For badly injured bats, the animals were euthanized first and then immediately swabbed to avoid any unnecessary stress or discomfort. This was done using a double swabbing technique to maximize DNA recovery (Pang & Cheung, 2007). A sterile swab (TS/8‐A, woodshaft with cotton tip, Technical Service Consultants) was moistened with 100 µl of molecular biology grade water immediately before use and then gently rolled and rotated over the surface of the bat wing at the site of the tear with an even and moderate pressure for approximately 10 s, and then the swab tip was placed in a sterile 1.5 ml tube. This was followed by swabbing the same bat wing area with a dry sterile swab, which was placed in a separate sterile 1.5 ml tube. Rehabilitators were also asked to complete a short questionnaire for each sample, providing information on species, age, and gender (if known). By September 2018, a total of 72 pairs of bat swabs had been provided by bat rehabilitators. This included 40 swabs from injured *P. pipistrellus* and 32 swabs from other UK bat species, which were as follows: eighteen soprano pipistrelle (*P. pygmaeus*), four whiskered bat (*Myotis mystacinus*), four brown long‐eared bat (*Plecotus auritus*), two Natterer's bat (*Myotis nattereri*) and one Serotine bat (*Eptesicus serotinus*), plus three swabs were from unknown bat species. All swabs were stored at −20°C prior to genetic analysis.

### 2.2 Information on bat wing tears

**FIGURE 1:**
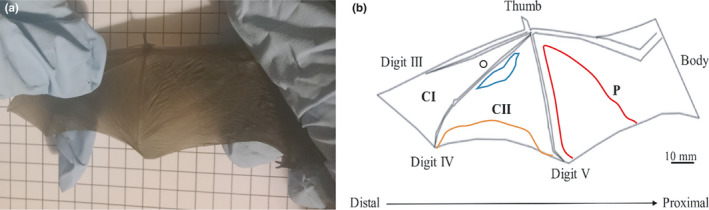
(a) Photograph of a bat wing held against gridded card; (b) image produced using Inkscape software showing the relative structure of the bat wing with the first chiropatagium (CI), second chiropatagium section (CII), and plagiopatagium (P) sections. Tear types are indicated: hole (black circle); contained tear (in blue); total tear (in red); and trailing edge tear (in orange)

Bat rehabilitators were asked to provide information on the nature of any wing injuries which were observed. Photographs of wing tears were taken soon after the bat was admitted to care, following swabbing. This was carried out on live adult animals during usual husbandry and rehabilitation procedures by the bat rehabilitators. The wing tears were photographed while the bat was awake (not during torpor, nor under anesthetic), and its wing was extended and held against 1 cm gridded card for scale (Figure 1a).

From the 72 samples that were submitted, 38 (52.7%) were received with images of the injured wings. Wing tears were traced from these images on to a bat wing diagram using Inkscape software. The position of wing tears was described in relation to the three largest sections of the wing (from distal to proximal): The first chiropatagium (CI) is the membrane between digits III and IV; the second chiropatagium section (CII) is the membrane between digits IV and V; and the plagiopatagium (P) which is the membrane between digit V and the body (Figure 1b). The tears in each of these sections were counted and categorized by four types: (a) *hole*—small puncture (usually < 2% of a wing section) (black circle, Figure 1b); (b) *contained tear*—elliptic tear resulting in loss of 5%–50% of the wing membrane (in blue, Figure 1b); (c) *total tear*—large tears running from the internal membrane to trailing edge, often affecting the bones (>50% of the membrane missing from the wing section) (in red, Figure 1b); and (d) *trailing edge tear*—horizontal tear only occurring at the trailing edge (in orange, Figure 1b) (Khayat et al., 2019). The frequency of tears in each section was normalized to the relative size of the wing section in each bat, which was extracted from tracing around each wing section in Inkscape.

Bat rehabilitators also volunteered information as to how the bat was found and the possible cause of the tear; bat rehabilitators emailed free‐text comments of the possible cause, describing any evidence for their decision. This information was received for 14 of the bat wing swab samples collected.

### 2.3 DNA extraction and quantification

DNA was extracted from both wet and dry swabs using an ISOLATE II Genomic DNA Kit (Bioline). First, 500 μl of phosphate‐buffered saline (Sigma‐Aldrich) was added to each swab in a 1.5 ml tube and vortexed for 2 min to remove cells from the swab. The swab was then removed, and 180 μl lysis buffer GL and 25 μl proteinase K solution were added. The standard manufacturer's protocol was then followed, with both wet and dry swab lysates added to the same spin column to collate samples. Cat whole blood samples were extracted using the same protocol, starting from the addition of lysis buffer GL and proteinase K solution.

DNA extracts were analyzed for concentration and purity using a NanoDrop™ 2000 Spectrophotometer (ThermoFisher).

### 2.4 Real‐time polymerase chain reaction (qPCR)

qPCR was used to analyze the bat wing swabs to determine the presence or absence of cat DNA. Primers and probes were designed using Primer‐BLAST, which included in silico PCR testing to ensure species specificity (https://www.ncbi.nlm.nih.gov/tools/primer‐blast/), and Eurofins qPCR Primer and Probe Design Tool (https://www.eurofinsgenomics.eu/en/ecom/tools/qpcr‐assay‐design/) software for amplification of the target sequence FCA749 locus of *F. catus* (Genbank accession number AY988149.1). This is a non‐gender‐specific marker with a predicted PCR product size of 248 bp, which aims to avoid potential issues related to DNA degradation of larger molecular weight targets in the samples tested.

Following optimization of the annealing temperature, primer, probe, and MgCl_2_ concentrations, samples were run using the following reaction conditions: 5 U/µl DNA polymerase (BIOTAQ DNA Polymerase, Bioline), 1 × reaction buffer (10× NH_4_ Reaction Buffer, Bioline), 25 mM dNTPs (dNTPs mix, Bioline), 1.5 mM MgCl_2_ (Bioline), 0.5 µM of forward primer (5′‐ATGCGTTCTCTGTCTCTC‐3′), 0.5 µM of the reverse primer (5′‐CATCTCACCGACCTAAAC‐3′), 0.25 µM probe (5′‐[HEX]‐TCACTGCTGGCCTCTTTCAAATCAC‐3′), and 5 µl of extracted DNA. All primer and probe sequences used throughout were obtained from Eurofins MWG Operon. Positive controls were prepared using DNA extracted from cat blood samples, along with negative controls containing no template DNA. Samples were run in triplicate on a MX3005P Real‐Time qPCR System (Agilent) with a thermal cycling profile of 94°C for 10 min, followed by 45 cycles of 94°C for 30 s, 56°C for 30 s, and 72°C for 30 s.

qPCR results were corroborated by running the amplified PCR products on a 2% agarose gel, made using 1× TBE buffer (0.1 M Tris base (Fisher Scientific), 0.1 M boric acid (Fisher BioReagents), and 0.02 M diaminoethanetetraacetic acid (EDTA) sodium salt (Fisher Scientific) in distilled water), and documented using a UV transilluminator (Geneflash Gel Documentation Darkroom, Syngene).

### 2.5 STR analysis

The samples which tested positive for cat DNA using the qPCR assay were then subject to STR profiling. Eleven microsatellite STR loci and a sex marker were used to generate the cat DNA profiles (Menotti‐Raymond et al., 2005). DNA amplification reactions were prepared using the following reagents in a 20 µl reaction volume: 5 U/µl DNA polymerase (BioTaq DNA polymerase, Bioline), 1× NH_4_ reaction buffer (Bioline), 25 mM dNTPs (dNTP mix, Bioline), 1.5 mM MgCl_2_, forward and reverse primers as described in (Menotti‐Raymond et al., 2005) in either single and multiplex format, and 4 µl of extracted DNA. Positive and negative controls were again included as for qPCR. The samples were then subjected to the following thermal cycling conditions on a Prime thermal cycler (Techne): initial denaturation at 90°C for 10 min; 35 cycles of 94°C for 1 min, 59°C for 1 min and 72°C for 1 min; and a final extension at 60°C for 45 min. Samples were then prepared for fragment analysis by adding 0.5 µl of PCR product to 0.3 µl of GeneScan™ LIZ 500 Size Standard (ThermoFisher Scientific) and 9.7 µl of Hi‐Di formamide (ThermoFisher Scientific) before running on a 3730 Genetic Analyzer (Applied Biosystems) at The Genomics Core Facility, University of Sheffield. Results were analyzed by using GeneMapper^®^ software (Version 3.7, ThermoFisher Scientific) to facilitate sizing of the observed alleles at each locus.

### 2.6 Statistical analysis

Statistical analysis was performed using SPSS version 24. A chi‐square test was used to compare: (a) the percentage of bats showing the presence of cat DNA in terms of species, gender, and age; (b) the percentage of tears that showed presence or absence of cat DNA; (c) the number of tears in each section of the wing; (d) the number of tears in each section normalized to the section area; and (e) the percentage of each type of tear (holes, contained tears, total tears, and trailing edge tears) between the samples with and without cat DNA present.

## 3 RESULTS

### 3.1 Presence of cat DNA

**FIGURE 2:**
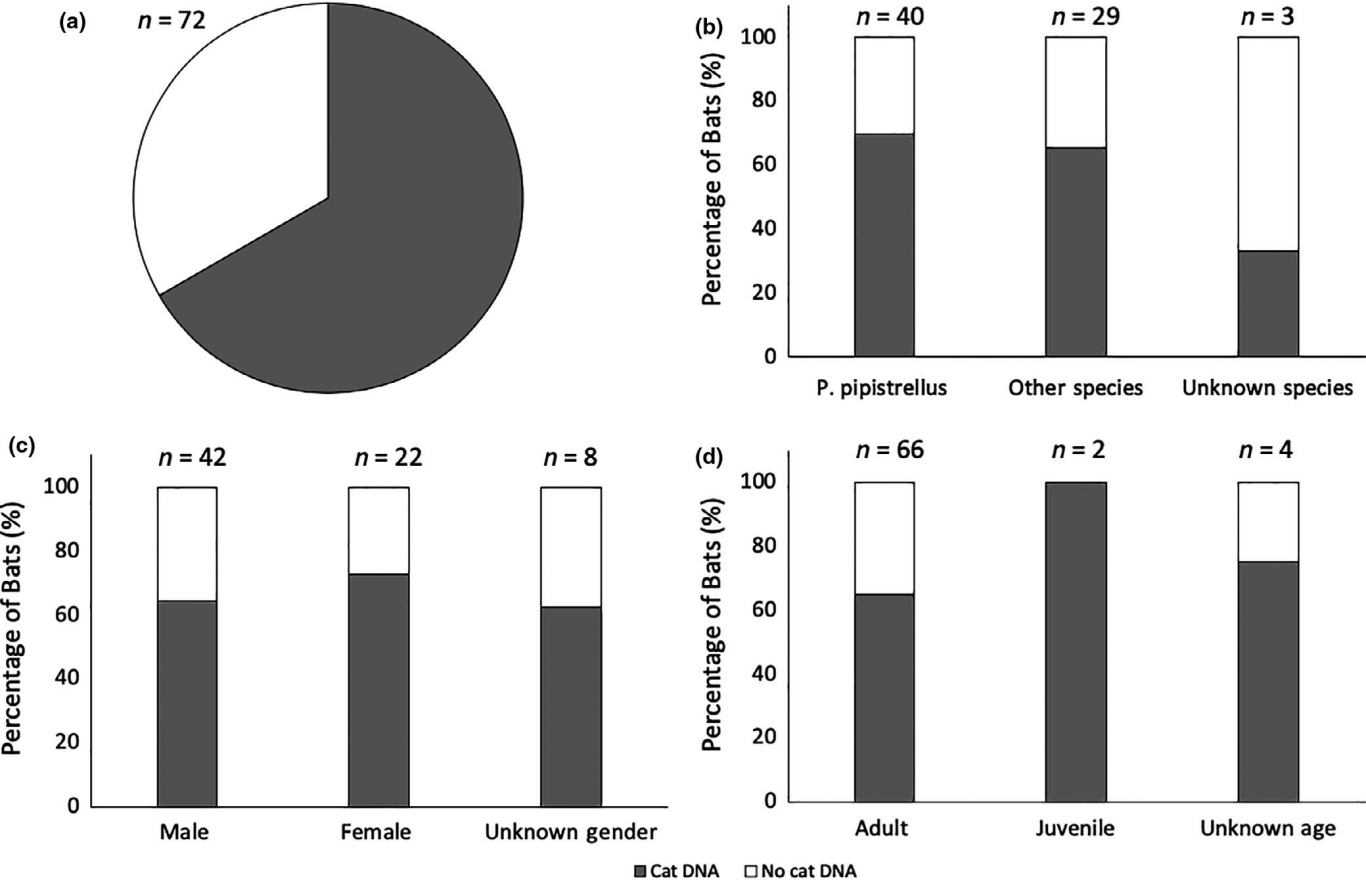
(a) Percentage of swab samples showing presence (gray) or absence (white) of cat DNA; (b) distribution of cat DNA with respect to UK bat species; (c) distribution of cat DNA with respect to bat gender; and (d) distribution of cat DNA with respect to the age of the bat

All the bat wing swab samples that were received from bat rehabilitators were quantified for DNA following DNA extraction. An average total DNA concentration of 1.2 ng/µl, with a range of 0.1–3.2 ng/µl, was observed. The samples were then analyzed for the presence of cat DNA using qPCR and confirmed using agarose gel electrophoresis. The results showed that 48 out of 72 (66.7%) of all samples were positive for the presence of cat DNA (Figure 2a). In order to assess for potential contamination, negative controls ran for DNA extraction and amplification were all clear. The Bat Care Guidelines (Miller, 2016) provided to all bat rehabilitators in the UK contains clear guidance about isolation of bats in their care, for example, separate gloves should be used when handling different bats and that any equipment should be sterilized after use. In addition, we were able to confirm with a number of our bat rehabilitators who provided us with samples (58%) that they did not own a cat. We compared the percentage of positive samples from this cohort (67%) with the total percentage of positive samples (66.7%) and found them to be in agreement.

The DNA results were then evaluated against the species, gender, and age of the bats. Cat DNA was present across all bat species sampled, with positive results obtained for 28/40 *P. pipistrellus*, 13/18 *P. pygmaeus*, 1/4 *M. mystacinus*, 1/4 *P. auritus*, 2/2 *M. nattereri*, 1/1 *E. serotinus*, and 1/3 of unknown bat species. There was no significant difference in the percentage of samples with cat DNA present in different bat species (*χ* ^2^ = 0.118, *df* = 1, *p* = .732) (Figure 2b). While there was no significant effect of gender on whether cat DNA was present (χ2 = 0.591, df = 1, p = .442), most of the swabs received here were from injured male bats (n = 42; Figure 2c). Age (juvenile or adult) had a marginal effect on whether wing swab samples had cat DNA present or not (*χ* ^2^ = 7.424, *df* = 1, *p* = .06), although this was not significant (*p* < .05) (Figure 2d).

### 3.2 STR profiling of cat DNA

**FIGURE 3:**
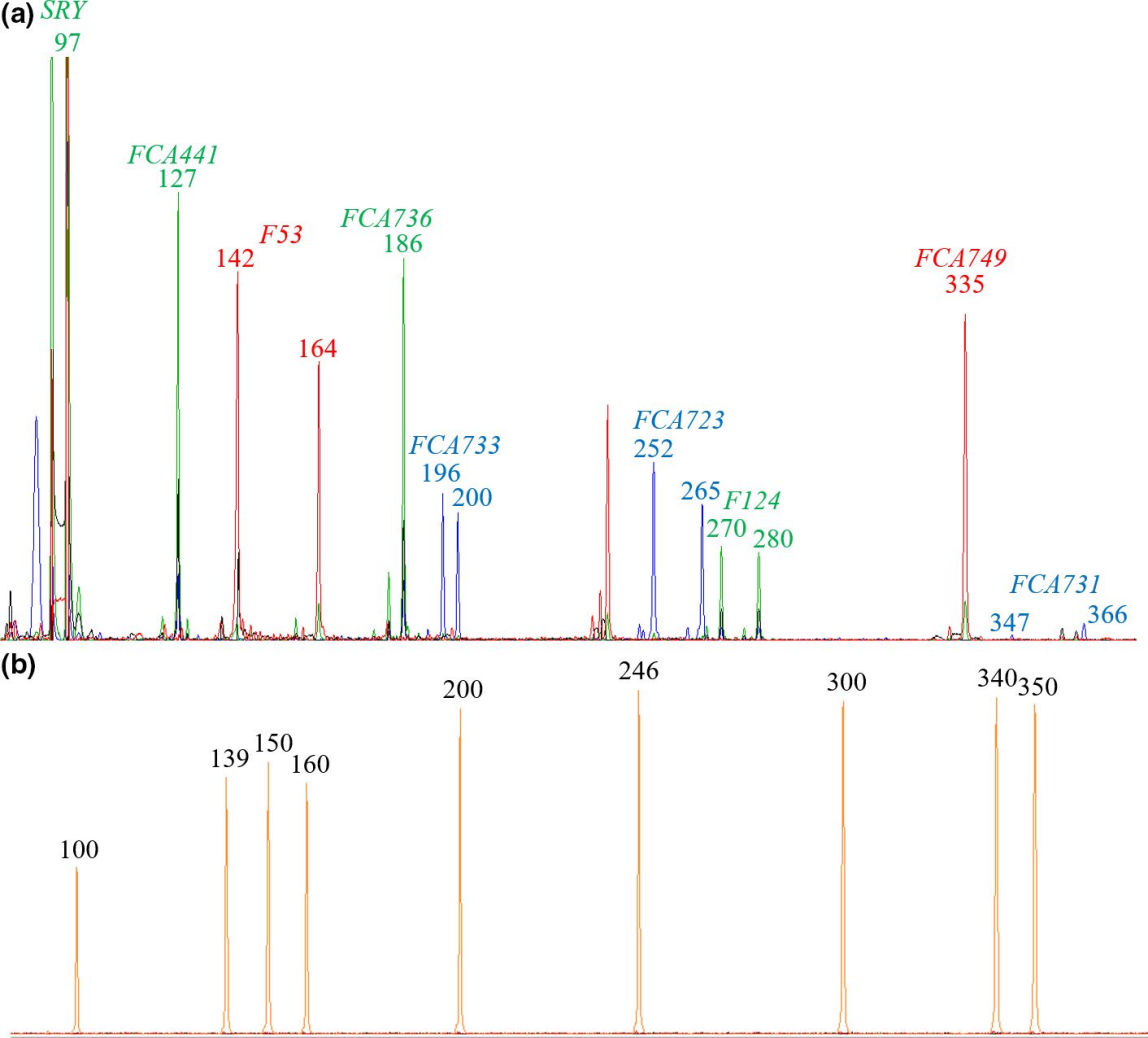
(a) Example of a full DNA profile produced from cat blood sample 01606; (b) example of a negative control sample showing the DNA size ladder, LIZ500 (orange peaks)

All samples which were positive for the presence of cat DNA using qPCR were then subject to STR profiling to gain further genetic information. DNA profiles were obtained from the positive control samples extracted from cat blood (example shown in Figure 3a) and the negative controls were clear of contamination (example shown in Figure 3b). For the bat wing swab samples, multiplex STR analysis yielded poor results with very few detectable alleles; therefore, STR analysis was then carried out using a single locus per amplification reaction and the results pooled. This produced better results, with partial DNA profiles detectable for 13 of the 48 samples. The majority of the alleles detected were for the lower molecular weight loci, with 85% less than 250 bp. The partial DNA profiles obtained from the bat wing swab samples were all compared to each other to determine whether any matches could be found, that is, whether or not the same cat may be responsible for the injuries sustained by the bats, but none were observed in our samples.

### 3.3 Comparison to wing tear information

**FIGURE 4:**
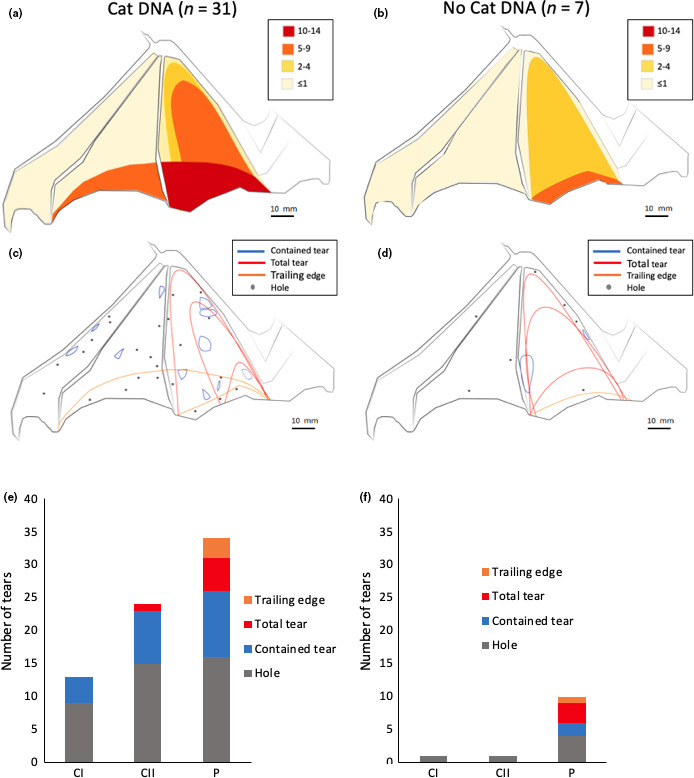
Evaluation of bat wing tears with respect to presence (left hand panels) or absence (right hand panels) of DNA. Panels a and b show the numbers of tears in each section of the wing. Panels c and d show all the wing tear positions of all tear types on each section of the wing. Panels e and f show the total numbers of different tear types in each wing section for the first chiropatagium section (CI), the second chiropatagium section (CII), and the plagiopatagium section (P)

Analysis of the number of tears and tear type, in relation to the presence or absence of cat DNA, showed that there was no significant difference in the number of tears (%) between the samples with and without cat DNA present (*χ* ^2^ = 1.922, *df* = 1, *p* = .166). For bat wing swabs where cat DNA was present, there were fewer tears in the CI section compared to the CII and P sections (*χ* ^2^ = 9.324, *df* = 2, *p* = .009) (Figure 4a). In the bat wing swab samples where no cat DNA was found, the P section of the wing had more tears than the CII and CI sections (*χ* ^2^ = 13.500, *df* = 2, *p* = .001) (Figure 4b). However, when the frequency of tears was normalized to the relative size of the sections, these differences were found to be not significant in either case (with cat DNA present: *χ* ^2^ = 0.0001, *df* = 2, *p* = 1; with cat no DNA present: *χ* ^2^ = 0.0001, *df* = 2, *p* = 1). There were significantly more total tears (%) (large tears running from the internal membrane to the trailing edge) in the bat samples with cat DNA present, compared to those without cat DNA (*χ* ^2^ = 8.758, *df* = 1, *p* = .003). The amount of other tear types (holes, contained tears, and trailing edge tears) did not differ significantly between the samples with cat DNA present and those without (all ps > 0.05) (Figure 4c–f).

The genetic data were also compared to the free‐text comments received from bat rehabilitators, where they were asked to suggest any possible causes of the bat wing injuries, and the reasons for this. When bat rehabilitators suspected cat involvement, cat DNA was found to be present in all but one case (92.9% of cases) (Table 1).

**TABLE 1:** Free‐text comments on suspected cause of bat wing injuries provided by bat rehabilitators, correlated with presence or absence of cat DNA

Free‐text comments	Cat DNA
Bat brought in by a cat	Absent
Householder has 2 cats, bat with older cat attack scars in wings. On this occasion, he was bitten left neck/ear and right shoulder. Signs of cat	Present
Householder has 2 cats—cat attack	Present
Householder has 2 cats—cat attack	Present
Owner of cat witnessed bat in cat's mouth	Present
Seen brought into home in cat's mouth	Present
Seen with cat circling around on ground	Present
Found grounded by dog. Injuries appear to be consistent with cat	Present
Householder has known roost in attic and owns 3 “well behaved” cats	Present
Property owner has 3 cats which catch birds often	Present
Cat at finder address	Present
Bad head wound as well as claw holes in wings	Present
Seen in cat's mouth	Present
Almost certainly cat damage	Present

## 4 DISCUSSION

### 4.1 Presence of cat DNA

Two‐thirds (48 out of 72) of the bat wing swab samples obtained showed the presence of cat DNA, a value higher than many previous reports based only on observational data, which suggests that cat predation can be more common than has been previously reported. For example, Ancillotto et al., (2013), reported cat involvement in an estimated 28.7% of bats being brought into rehabilitation centers, and Mühldorfer, Speck, Kurth, et al. (2011) demonstrated that cat predation accounted for 19.5% of bat deaths based on postmortems. A recent study by Vlaschenko et al. (2019) reported a similar result that 68% of the common noctule (*Nyctalus noctule*) bats found dead were killed by cat predation during the winter when bats hibernated in a large concrete building. It is hypothesized that while qPCR is a very sensitive technique for the detection of cat DNA, this value of two‐thirds of bats being predated upon by cats could still be an underestimation, due to factors such as (a) bats not always being brought to the attention of rehabilitators; (b) bats not having visible wing tears or having sustained injuries to the torso which are sometimes not apparent (e.g., hidden under fur) when first admitted into care; (c) insufficient DNA quantity transferred from cat to bat during the predation event; and (d) potential variability in the swabbing technique and sample storage by participating bat rehabilitators.

Bat species type, gender, and age were shown to have no effect on whether or not cat DNA was present on the wing swab samples. All the bat species examined here (*P. pipistrellus* and other UK species) use similar roost types, and may all use buildings (BCT, 2019; Boughey, Lake, Haysom, & Dolman, 2011) in rural and semi‐urban areas, which could mean encountering free‐ranging cats at a similar rate (Ancillotto et al., 2013). Previous observational studies have reported more cat attacks on female bats than males, particularly during the summer which can threaten reproductive colonies (Ancillotto et al., 2013; Vlaschenko et al., 2019), although we had more males within our samples. In agreement, we observe that more males are usually admitted to care than females; a survey of all our records over the previous 4 years has found that there are consistently more males in care than females (52%–64% admissions are male), although this is not a significant difference (*p* > .05). Other studies have reported that juveniles and adults are just as likely to be targeted by cat attacks (Ancillotto et al., 2013; Vlaschenko et al., 2019), which is supported by our data.

### 4.2 STR profiling of cat DNA

Forensic DNA analysis was possible on the bat wing swab samples; however, only partial STR profiles were produced. Partial STR profiles can occur with low template DNA concentrations as a result of allelic dropout or peak imbalance. Such profiles can also arise when using low quality DNA, where degradation has happened which prevents amplification of larger loci due to DNA fragmentation (Butler, 2001), which follows the patterns observed in this study. Several factors could have contributed to DNA degradation in the bat wing swab samples such as the amount of time in between the predation event and the bat wing being swabbed and potential delays in posting by the bat carers.

Multiple samples were obtained from nearby geographical locations, up to a maximum of 30 miles, to specific bat carers (i.e., 17 samples from Kent and 24 samples from Dorset) (Trust, 2020). The relative percentage of samples showing the presence of cat DNA was mapped to location (see Figure S1). As it is not uncommon for bat carers to report the same cat presenting bats in successive years, there was the possibility that the same cat may be responsible for more than one bat interaction. Although no DNA profile matches were observed here, a study in mainland Southwest Finland, proposed the idea of “super predator cats", where just six cats (9%) accounted for 40% of all observed captures in or around the city of Turku, with no difference between male and female cats (Kauhala et al., 2015). This supports the principle of prey specialization in cats; identifying individual cats which specifically target species of conservation concern offers a potential means of reducing the effect of cat predation on wildlife (Dickman & Newsome, 2015). The ability to obtain DNA profiling results from bat wing swabs enables us to identify individual cats which repeatedly prey on bats without, or in support of, observational data. This could then be used to inform the development of control methods that specifically focus on those individuals (Hardman, Moro, & Calver, 2016; Moseby et al., 2015).

### 4.3 Comparison to wing tear information

Bat carers are able to reliably identify tears caused by cats (93% agreement), and larger tears were present on the wings with cat DNA present. However, it is challenging to identify cat predation solely by looking at the tear type and position. No specific tear type was associated with the presence of cat DNA, and although more tears were found in the P section overall (Figure 4), when the size of each wing section was controlled for, each section had the same amount of tears. The P section is the largest section of the wing so it is likely to receive more tears than the most distal section of the wing CI. It may be that some samples with cat DNA present are not being detected by the method (e.g. due to swabbing procedures and storage time), which makes it challenging to associate tear descriptions with the presence of cat DNA. Khayat et al. (2019) found more tears in the P section of the wing and suggested that cats specifically target the bat's body, causing many tears in the P section; our results do not confirm this.

Many other causes, apart from cat predation, are likely to cause wing tears, especially on the P section. While collisions are probably more likely to affect the distal sections of the wing, perhaps injuries during take‐off (when the wing unfurls) or grounding might be a possible cause of these tears in the P section. Certainly, this section is extended first before flight and might get caught or snagged during flight preparation (Gardiner, Dimitriadis, Codd, & Nudds, 2011). Due to its position closer to the body, the P section might also be more likely to snag on branches during hanging, or be less manoeuvrable to tuck in to escape collisions. Vegetation, such as brambles or other thorns, being present at take‐off and landing sites is a likely cause of many tears but hard to identify objectively, whereas detection of other potential predators such as ferrets could also be achieved through genetic analysis.

### 4.4 Impact of cat predation

Free‐roaming domestic cats cause a significant number of bird and mammal fatalities and, with the number of cats increasing annually (Woods et al., 2003), the effect of cat predation on wildlife is likely to rise. Therefore, the number of injured bats from cat attacks will likely increase in the future. Previous research which has examined cat predation on wildlife, especially mammals and birds, has led to recommendations to reduce predation, and these are also pertinent for reducing bat predation. Recommendations include the following: (a) being mindful when making residential developments in close proximity to protected species habitats (Phillips et al., 2001); (b) night‐time curfews for domestic cats (Barratt, 1997); and (c) use of bells (Ancillotto et al., 2013) or novel cat collars such as Birdsbesafe^®^, which have been shown to be effective in reducing bird predation (Willson, Okunlola, & Novak, 2015).

As well as causing wing tears, cat attacks can also lead to bacterial diseases in bats (Mühldorfer, Speck, & Wibbelt, 2011), which can be transmitted to bats from cat saliva (Mühldorfer, Speck, Kurth, et al., 2011). Cat claws carry bacteria which is also likely to transfer to the bat upon contact, in a similar manner to cat‐scratch disease that can be acquired by humans upon injury (Christina, Shubhayu, & Paul, 2016; Kirkpatrick & Glickman, 1989). Cats may also receive a viral infection from the bats, such as Nipah virus (NiV) and European bat lyssaviruses (EBLVs), which could lead to cat mortality (Dacheux et al., 2009; Epstein et al., 2006). Therefore, investigating the interactions of cats and bats can have important implications for both species.

## 5 CONCLUSIONS

This study demonstrates that forensic DNA analysis techniques can be used to evaluate the possibility of cat predation upon bats, as an alternative to observational data. By swabbing the site of bat wing tear injuries, the presence of cat DNA can be identified. Furthermore, when cat DNA is present, it is also possible to obtain (at least a partial) DNA profile from the individual. The results presented here suggest that cat predation on bats, at least in the UK, may be responsible for more than two‐thirds of admissions to bat rehabilitators. Therefore, a better understanding of cat and bat interactions has implications for both cat and bat populations, as well as their health and welfare. For future work, this could be combined with looking at larger sample sizes and monitoring of the release outcomes from injuries sustained through cat predation. Monitoring of samples over a longer period of time, with higher numbers of bat casualties, may result in cat DNA profile matches being obtained. In addition, investigating the seasonality of wing tear casualties and associating this with the sex of the bats would help us to understand the effect of wing tears on bat population demographics and reproduction. This would allow us to better predict the extent of the problem, and the long‐term effects on bat populations.

## CONFLICT OF INTEREST

The authors declare that they have no conflicts of interest to report.

## AUTHOR CONTRIBUTIONS

**Rana O. S. Khayat:** Data curation (equal); formal analysis (equal); writing – original draft (equal); writing – review and editing (equal). **Robyn A. Grant:** Conceptualization (equal); supervision (equal); writing – original draft (equal); writing – review and editing (equal). **Hazel Ryan:** Resources (equal); writing – review and editing (equal). **Louise M. Melling:** Supervision (equal); writing – original draft (equal); writing – review and editing (equal). **Gary Dougill:** Supervision (equal); writing – original draft (equal). **David R. Killick:** Resources (equal); writing – review and editing (equal). **Kirsty J. Shaw:** Conceptualization (equal); supervision (equal); writing – original draft (equal); writing – review and editing (equal).

## Supporting information

Figure S1

Figcap

## Data Availability

Data are available on Dryad: https://doi.org/10.5061/dryad.zcrjdfn88.
