# Supplementary figures and images for: Investigating cat predation as the cause of bat wing tears using forensic DNA analysis
Source: Ecol Evol. 2020 Jul 6;10(15):8368–78. doi: 10.1002/ece3.6544 (PMC7417221; doi:10.1002/ece3.6544)

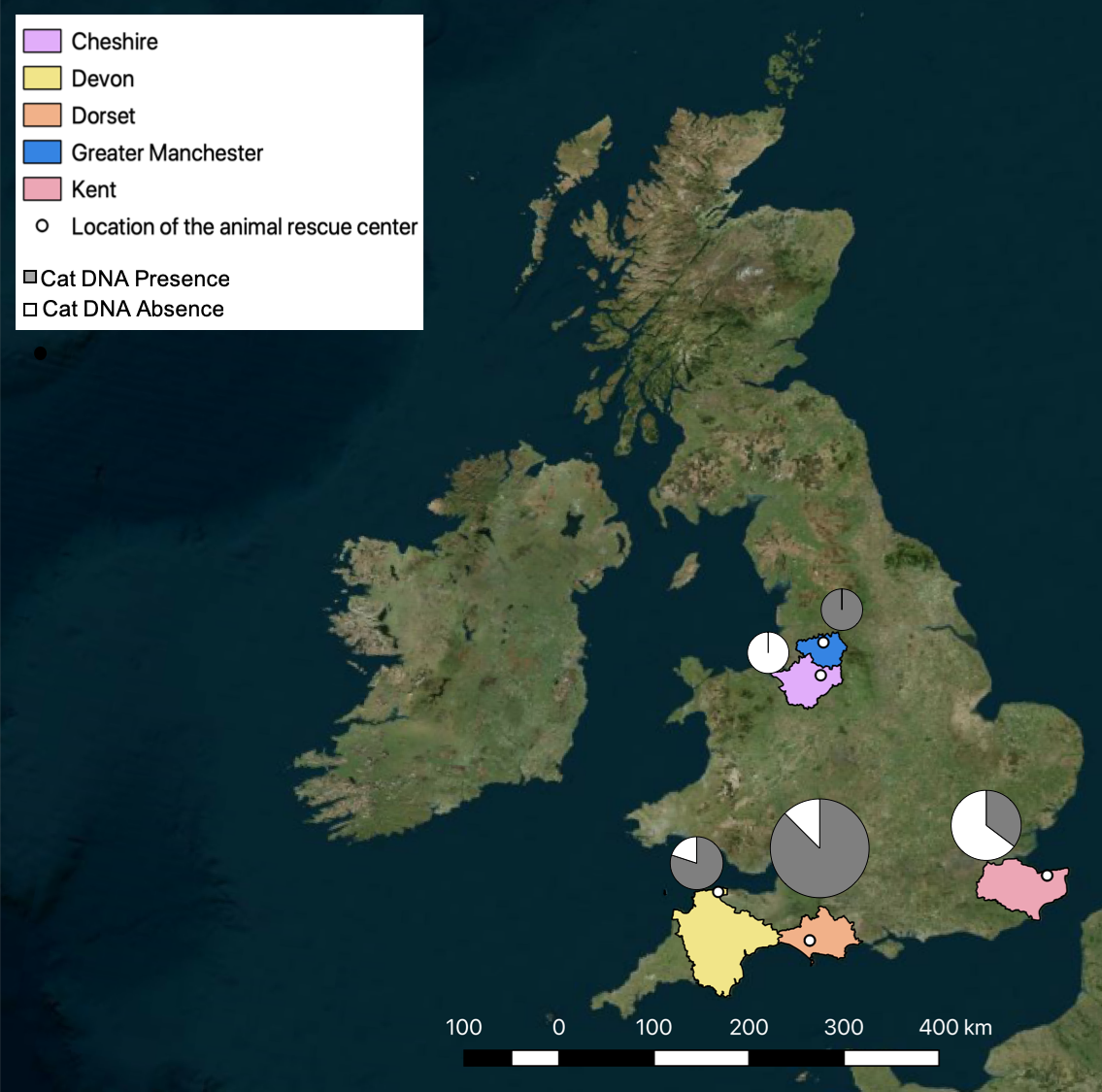

Supplement: Supplementary file 1 — Figure S1 [file ECE3-10-8368-s001.tiff]
