# Supplementary material for: Investigating cat predation as the cause of bat wing tears using forensic DNA analysis
Source: Ecol Evol. 2020 Jul 6;10(15):8368–78. doi: 10.1002/ece3.6544 (PMC7417221; doi:10.1002/ece3.6544)
Supplement: Supplementary file 2 — Figcap [file ECE3-10-8368-s002.docx]

**Figure S1:** Map of the United Kingdom showing where the bat wing swab samples were received from, and the percentage of positive and negative samples for cat DNA at each location. The size of the pie chart represents the relative sample size. The image was created using QGIS3 software (version 3.2.2) (<https://www.qgis.org/en/site/>)
